# Avalanche Survival Rates in Switzerland, 1981-2020

**DOI:** 10.1001/jamanetworkopen.2024.35253

**Published:** 2024-09-25

**Authors:** Simon Rauch, Hermann Brugger, Markus Falk, Benjamin Zweifel, Giacomo Strapazzon, Roland Albrecht, Urs Pietsch

**Affiliations:** 1 Institute of Mountain Emergency Medicine, Eurac Research, Bolzano, Italy; 2 Department of Anesthesia and Intensive Care Medicine, Hospital of Merano, Merano, Italy; 3 International Commission of Mountain Emergency Medicine, Zurich-Kloten, Switzerland; 4 WSL Institute for Snow and Avalanche Research, Davos, Switzerland; 5 Department of Medicine, University of Padova, Padova, Italy; 6 Division of Perioperative Intensive Care Medicine, Cantonal Hospital St Gallen, St Gallen, Switzerland; 7 Department of Emergency Medicine, Bern University Hospital (Inselspital), University of Bern, Bern, Switzerland; 8 Swiss Air-Ambulance (Rega), Zurich, Switzerland

## Abstract

**Importance:**

Survival probability among individuals critically buried by avalanche is highly time dependent, which was demonstrated 30 years ago. However, it remains unclear whether avalanche survival probability has changed over time.

**Objective:**

To assess the avalanche survival rate and probability as well as the rescue probability over the past 4 decades.

**Design, Setting, and Participants:**

In this cohort study, avalanche data from Switzerland that were collected by the WSL Institute for Snow and Avalanche Research (SLF) in Davos were analyzed from the winter beginning in 1981 to that beginning in 2020 and compared with data from the period 1981 to 1990. Data were analyzed from January to April 2024.

**Exposure:**

Critical avalanche burial (ie, burial involving the head and chest).

**Main Outcomes and Measures:**

Survival rate among individuals critically buried by avalanche, survival probability, and rescue probability in relation to time buried under the avalanche.

**Results:**

The study included 1643 individuals critically buried by avalanche (mean [SD] age, 37 [13.7] years; 1090 of 1342 with known sex [81.2%] were male) among 3805 avalanches involving 7059 persons. Compared with the period from 1981 to 1990, the total survival rate over the full study period increased from 43.5% (95% CI, 38.8%-48.3%) to 53.4% (95% CI, 51.0%-55.8%). Survival probability remained high at 91% (95% CI, 80%-100%) for rescue during the first 10 minutes but then decreased to 31% (95% CI, 11%-51%) for rescue between 10 and 30 minutes. The survival rate among those buried long term (>130 minutes) increased from 2.6% (95% CI, 0.7%-6.9%) to 7.3% (95% CI, 4.8%-10.7%). The median rescue time decreased from 45 (IQR, 15-148) minutes to 25 (IQR, 10-85) minutes. Survival rates among individuals rescued from avalanche by organized rescue teams increased from 14.0% (28 of 200) to 22.9% (161 of 704).

**Conclusions and Relevance:**

This cohort study of 1643 individuals critically buried by avalanche found that over the past 4 decades, total survival rates considerably increased and rescue times decreased. Survival rates among those buried long term (>130 minutes) also increased. These findings are likely attributable to collaborative efforts among stakeholders to enhance avalanche search-and-rescue techniques and medical interventions.

## Key Points

**Question:**

Have survival rates, survival probability, and rescue probability for individuals critically buried by avalanche changed over the past 4 decades?

**Findings:**

In this cohort study of 1643 individuals critically buried by avalanche in Switzerland comparing the period of winters beginning in 1981 to 1990 with those beginning 1981 to 2020, the total survival rate increased, the probability of survival if rescued within 10 minutes remained high but between 10 and 30 minutes decreased, survival after 130 minutes of burial improved, and median rescue time decreased.

**Meaning:**

These results suggest that improved avalanche search-and-rescue techniques and advancements in medical treatment have been successful but the risk of early suffocation remains substantial.

## Teaser

This cohort study of individuals critically buried by avalanche in Switzerland investigates changes in survival rates and probability of survival and rescue from winter 1981-1982 to winter 2020-2021.

## Introduction

Survival probability for individuals critically buried in avalanches (ie, involving the head and chest) is highly time dependent. Falk et al^1^ provided the first rigorous statistical analysis of this relationship 30 years ago. The avalanche survival chances were calculated using a nonparametric estimation procedure with double-censored data,^2^ including all avalanche accidents in Switzerland between 1981 and 1991. Of 422 critically buried individuals, 241 (57%) were found dead on extrication. However, within the first 15 minutes of burial, only 8 of 123 rescued individuals (7%) were found dead (2 from asphyxia and 6 from fatal trauma), resulting in a survival probability of 92% during the first 15 minutes. The survival probability then decreased sharply to just 30% at 35 minutes after burial, primarily due to acute asphyxia. Deaths between 35 and 130 minutes were attributed to a combination of slow asphyxia and hypothermia. Long-term survival beyond 130 minutes was poor at only 2.6%. This time-dependent survival probability highlighted the critical importance of rapid and efficient search-and-rescue efforts, particularly by uninjured companions, as organized rescue teams often face limited success due to these time constraints.

This study aimed to clarify whether avalanche survival rates and probabilities have changed over time. Its primary objective was to evaluate the survival rates, survival probabilities, and rescue probabilities for individuals critically buried by avalanche over the past 4 decades.

## Methods

In this cohort study, avalanche data from Switzerland were collected by the WSL Institute for Snow and Avalanche Research (SLF) in Davos, including detailed records of each event. These data cover date, time, burial duration, type of rescue (self-rescue, companion rescue, or organized rescue service), and survival status (alive or deceased as the final outcome). For this study, we analyzed data from the winter beginning in 1981 to that beginning in 2020 and compared them with data from winters beginning in 1981 to 1990, focusing exclusively on individuals critically buried by avalanche. The institutional review board of the Cantonal ethics committee (EKOS) reviewed the study design and granted permission for the use of patient data without individual consent according to the federal act on research involving human beings and the ordinance on human research with the exception of clinical trials. The permission covers the use of anonymized patient data. This study followed the Strengthening the Reporting of Observational Studies in Epidemiology (STROBE) reporting guideline.^3^

### Statistical Analysis

Continuous variables are given as means and SDs or medians and IQRs, as appropriate, and categorical variables as frequencies and percentages. The 95% CIs for proportions are Jeffreys intervals and are 2-sided. Groups were compared using the Kruskal-Wallis rank sum test for continuous variables and the χ^2^ test for categorical variables and were analyzed in SPSS, version 29 (IBM Corp). Missing rescue times were imputed via inverse transform sampling, conducted separately for each rescue type and distinctly for survivors and nonsurvivors (the eMethods, eTable, and eFigures 1-3 in Supplement 1 give a detailed description). Survival analyses were performed using R, version 4.4.1 (R Project for Statistical Computing) and its packages survival, interval, survminer, and icenReg. Survival was estimated using the Turnbull algorithm for interval-censored data^4^ using the function survival::survfit and plotted with survminer::ggsurvplot. Individuals extricated alive were right censored, whereas nonsurvivors were left censored. Survival probability refers to the likelihood of survival as depicted by the survival curve, while survival rate is the final outcome after extrication. Comparisons between groups were completed using a log-rank–type test for interval-censored data using the function icenReg::ic_sp or the log-rank test for right-censored data. All tests were 2-sided, and *P* < .05 was considered statistically significant. Data were analyzed from January to April 2024.

## Results

**Figure 1.**
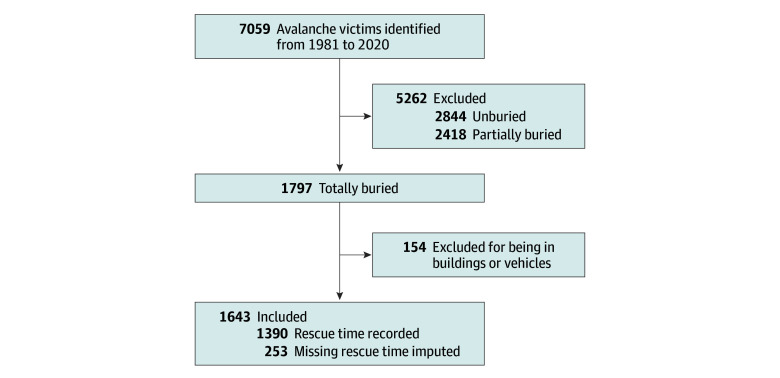
Flowchart of Avalanche Survivors and Nonsurvivors for Analysis

A total of 1643 individuals critically buried by avalanche (mean [SD] age, 37 [13.7] years; 252 of 1342 with known sex [18.8%] were female and 1090 [81.2%] male) were included among 3805 avalanches involving 7059 persons. The characteristics of the included individuals are shown in the Table. In 253 of these 1643 cases (15.4%), burial duration data were missing, with 26.1% of data missing for survivors (229 of 878) and only 3.1% for nonsurvivors (24 of 765) (χ^2^ test *P* < .001) (Figure 1). Among the 253 cases with missing burial duration data, 176 individuals (69.6%) were rescued by companions or self-rescued, while only 34 (13.4%) were rescued by organized rescue teams (χ^2^ test *P* < .001). Since missing times are not random and thus carry information, imputation was necessary to prevent bias.

**Table.** Characteristics of Avalanche Survivors and Nonsurvivors in the Different Periods

Characteristic	Individuals^a^					*P* value^b^	*P* value for trend
	Total (N = 1643)	1981-1990 (n = 416)	1991-2000 (n = 349)	2001-2010 (n = 469)	2011-2020 (n = 409)		
Activity							
Backcountry skiing^c^	1100 (67.0)	298 (71.6)	226 (64.8)	307 (65.5)	269 (65.8)	.13	.09
Out-of-bounds skiing^d^	543 (33.0)	118 (28.4)	123 (35.2)	162 (34.5)	140 (34.2)		
Survivors							
Overall	878 (53.4)	181 (43.5)	191 (54.7)	276 (58.8)	230 (56.2)	<.001	<.001
By rescue time, min							
0-15	600/662 (90.6)	115/125 (92.0)	121/127 (95.3)	194/221 (87.8)	180/189 (95.2)	.13	.19
16-30	141/257 (54.9)	30/50 (60.0)	38/67 (56.7)	37/67 (55.2)	36/73 (49.3)	.67	.23
31-130	115/424 (27.1)	33/127 (26.0)	26/79 (32.9)	35/122 (28.7)	21/96 (21.9)	.41	.52
>130	22/300 (7.3)	3/114 (2.6)	6/76 (7.9)	10/59 (16.9)	3/51 (5.9)	.008	.06
By type of rescue							
Companions	604/808 (74.8)	134/197 (68.0)	130/169 (76.9)	184/234 (78.6)	156/208 (75.0)	.07	.09
Organized rescue service	161/704 (22.9)	28/200 (14.0)	42/158 (26.6)	57/199 (28.6)	34/147 (23.1)	.003	.02
Nonsurvivors	765 (46.6)	235 (56.5)	158 (45.3)	193 (41.2)	179 (43.8)	<.001	<.001
Rescuer							
Companions	808 (49.2)	197 (47.4)	169 (48.4)	234 (49.9)	208 (50.9)	<.001	<.001
Organized rescue service	704 (42.8)	200 (48.1)	158 (45.3)	199 (42.4)	147 (35.9)		
Self-rescue	57 (3.5)	18 (4.3)	13 (3.7)	13 (2.8)	13 (3.2)		
Unknown	74 (4.5)	1 (0.2)	9 (2.6)	23 (4.9)	41 (10.0)		
Burial time							
Known	1390 (84.6)	389 (93.5)	309 (88.5)	371 (79.1)	321 (78.5)	<.001	<.001
Missing							
Total	253 (15.4)	27 (6.5)	40 (11.5)	98 (20.9)	88 (21.5)	<.001	<.001
Survivors	229/253 (90.5)	26/27 (96.3)	35/40 (87.5)	91/98 (92.9)	77/88 (87.5)	.39	.31
Nonsurvivors	24/253 (9.5)	1/27 (3.7)	5/40 (12.5)	7/98 (7.1)	11/88 (12.5)		
Burial time, median (IQR), min							
Rescue by companions	10 (5-20)	15 (8-30)	10 (5-24)	10 (5-15)	10 (5-20)	.03	<.001
Organized rescue service	90 (41-515)	153 (70-855)	112 (40-600)	60 (35-225)	65 (35-420)	.03	
Self-rescue	5 (3-10)	5 (3-15)	5 (1-9)	3 (3-8)	2 (1-5)	.40	
Total	25 (10-85)	45 (15-148)	30 (10-105)	20 (9-55)	20 (10-45)	<.001	
Depth of burial, median (IQR), cm							
Rescue by companions	55 (30-100)	53 (40-100)	50 (30-100)	50 (30-100)	68 (30-100)	.08	<.001
Organized rescue service	100 (60-150)	100 (70-170)	100 (55-150)	100 (58-150)	100 (50-160)	.57	
Self-rescue	28 (10-40)	40 (30-50)	10 (10-30)	20 (15-30)	10 (10-20)	.003	
Total	80 (50-140)	80 (50-145)	80 (40-135)	80 (40-135)	80 (40-150)	.49	

^a^ Data are presented as number or number/total number (percentage) of participants unless otherwise indicated.

^b^ The χ^2^ test was used for categorical data; the Kruskal-Wallis test was used for continuous variables.

^c^ Backcountry skiing includes any activity that takes place in the backcountry, away from lift-serviced ski areas.

^d^ Out-of-bounds skiing involves the access to uncontrolled terrain beyond the boundaries of ski area.

### Survival Rate and Survival Probability in Relation to Duration of Critical Burial

**Figure 2.**
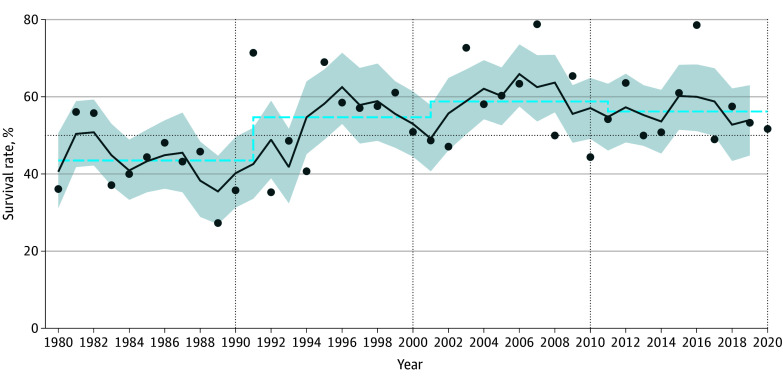
Survival Rates by Single Year, Running 3 Years, and Decade Data points indicate single years; dark blue line, running 3 years; dashed blue line, decade; shading, 95% CIs. Vertical dotted lines represent decade cutoffs and the horizontal dotted line, the 50% case survival rate. The number of individuals was 416 between 1981 and 1990, 349 between 1991 and 2000, 469 between 2001 and 2010, and 409 between 2011 and 2020.

**Figure 3.**
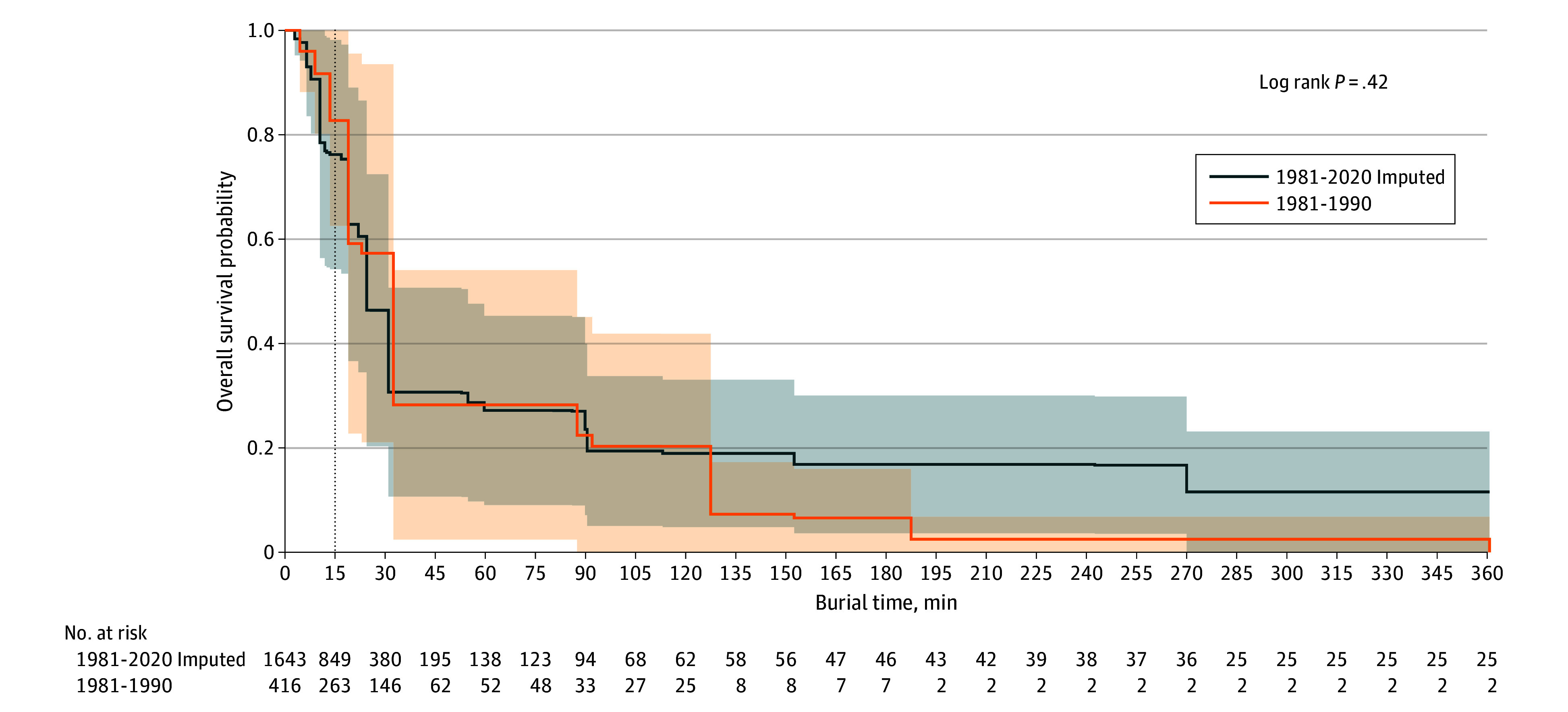
Cumulative Probability of Survival by Time Buried Under Avalanche in Minutes Dark blue line represents new data from 1981 to 2020, with missing times imputed; orange line, data from 1981 to 1990 as used in the study by Falk et al,^1^ with missing times imputed; vertical dotted line, 15-minute reference mark.

The total survival rate increased by 9.6 percentage points, from 43.5% (95% CI, 38.8%-48.3%) in the period from 1981 to 1990 to 53.4% (95% CI, 51.0%-55.8%) in the overall period (1981-2020) (χ^2^ test *P* < .001) (Figure 2). The total survival probability relative to the duration of critical burial (ie, the survival curve) did not significantly change over the past 40 years (hazard ratio for 1981-1990 vs 1981-2020: 1.07; 95% CI, 0.89-1.27; *P* = .42) (Figure 3). However, 2 key differences emerged when comparing the 2 survival curves. First, the survival probability decreased earlier in the 1981-2020 curve, occurring after 10 minutes of burial compared with after 15 minutes in the 1981-1990 curve. Between 1981 and 2020, the survival probability after 10 minutes of burial was 91% (95% CI, 80%-100%), decreasing to 76% (95% CI, 54%-98%) after 15 minutes. In comparison, from 1981 to 1990, survival probability was 92% (95% CI, 80%-100%) at 10 minutes and remained comparatively high at 83% (95% CI, 62%-100%) after 15 minutes. After 30 minutes of burial, the survival probability dropped to 31% (95% CI, 11%-51%) between 1981 and 2020 and to 28% (95% CI, 2%-54%) between 1981 and 1990.

The second difference was in the survival rate for long-term burial (beyond 130 minutes), which increased from 3 of 114 (2.6%; 95% CI, 0.7%-6.9%) between 1981 and 1990 to 22 of 300 (7.3%; 95% CI, 4.8%-10.7%) between 1981 and 2020 (χ^2^ test *P* = .008). The survival rate for individuals rescued by companions improved from 68.0% (134 of 197) between 1981 and 1990 to 74.8% (604 of 808) between 1981 and 2020 (χ^2^ test *P* = .07). For individuals rescued by organized rescue teams, the survival rate increased from 14.0% (28 of 200) between 1981 and 1990 to 22.9% (161 of 704) between 1981 and 2020 (χ^2^ test *P* < .001).

### Probability of Rescue in Relation to Burial Time

**Figure 4.**
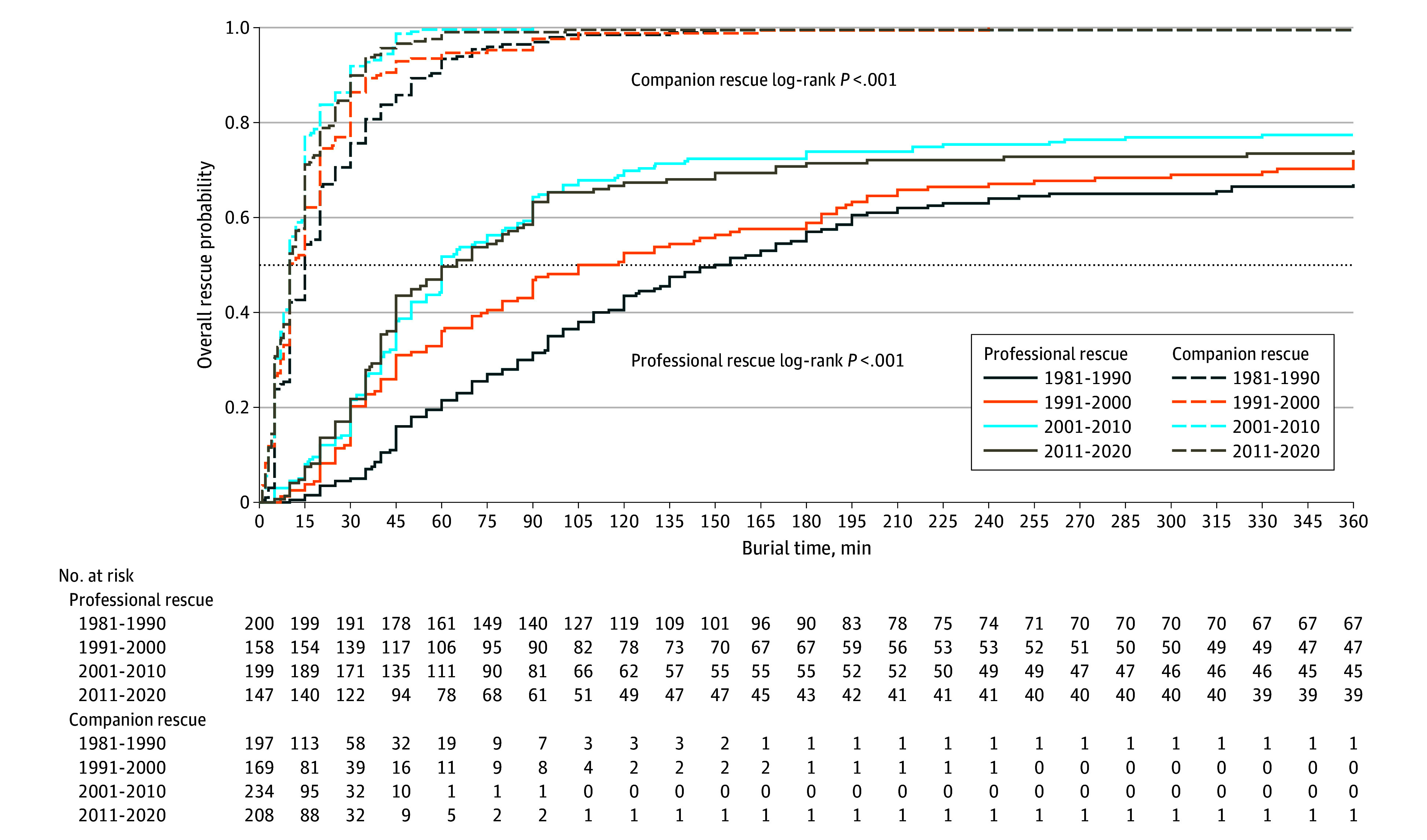
Probability of Rescue by Rescue Type and Time Buried Under Avalanche in Minutes Horizontal dotted line indicates rescue probability of 50%.

The median rescue time (ie, the time from burial to extrication) significantly decreased from 45 minutes (IQR, 15-148 minutes) between 1981 and 1990 to 25 minutes (IQR, 10-85 minutes) between 1981 and 2020 (log-rank test *P* < .001), resulting in a notable reduction of 44.4% in burial duration (Figure 4). Specifically, the median rescue time for individuals rescued by companions decreased from 15 minutes (IQR, 8-30 minutes) to 10 minutes (IQR, 5-20 minutes) (log-rank test *P* < .001), while for those rescued by organized services, it decreased from 153 minutes (IQR, 70-855 minutes) to 90 minutes (IQR, 41-515 minutes) (log-rank test *P* < .001). Comparing the periods 1981 to 1990 with 1981 to 2020, the rate of companion rescues increased from 47.4% (197 of 416) to 49.2% (808 of 1643), whereas the rate of professional rescues decreased from 48.1% (200 of 416) between 1981 and 1990 to 42.8% (704 of 1643) between 1981 and 2020 (χ^2^ test *P* < .001). Individuals rescued by companions had higher chances of survival compared with those rescued by organized rescue teams (relative risk, 2.9; 95% CI, 2.6-3.3).

## Discussion

Our study revealed 3 key findings. First, the total avalanche survival rate increased over the past 4 decades. Second, while the survival probability relative to the duration of critical burial remained unchanged, asphyxia onset appeared to be earlier than previously assumed and long-term burial survival rates improved. Third, the median rescue time decreased.

When comparing the survival curves from 1981 to 1990 with those from 1981 to 2020, a notable difference within the first 15 minutes of burial emerged. Although the survival probability exceeded 90% within the first 10 minutes of burial in both periods, it dropped to 76% after 15 minutes between 1981 and 2020, compared with 83% in the 1981-1990 period. The previously assumed high survival probability up to 15 minutes after burial^1^ was not supported by the extended dataset with imputed missing rescue times used in our study (eMethods in Supplement 1). The shift in the inflection point of the survival curve from 15 minutes to 10 minutes (Figure 3) may be attributed to either severe trauma or an earlier onset of asphyxia, both of which impact the initial stages of the survival curve.^5^ Haegeli et al^5^ conducted a comparative analysis of avalanche survival patterns between Canada and Switzerland, presenting distinct survival curves for different Canadian snow climates, including 1 specifically accounting for asphyxia-related deaths while excluding trauma fatalities. The curves for denser snow climates and those focusing solely on asphyxia showed a noticeable decline in survival rates starting as early as 10 minutes after burial, similar to our findings from 1981 to 2020. This suggests that the shift in the inflection point from 15 minutes (1981-1990) to 10 minutes (1981-2020) observed in our data are more likely due to an earlier onset of asphyxia than to an increase in trauma-related fatalities. The reason for this remains unclear, but it may be hypothesized that changes in snow density over time could be a factor, as higher snow density is associated with faster onset of asphyxia.^6^ However, to our knowledge, there is currently no strong evidence to support this hypothesis. In this study, the risk of dying from suffocation between 10 and 30 minutes of burial remained unchanged over the past 4 decades, highlighting the greatest challenge in the search and rescue of individuals critically buried by avalanche. The time window for a successful rescue is short and should be considered by all educators, stakeholders, and manufacturers of avalanche safety equipment.

In contrast to short burials, the survival rate after long-term burial (>130 minutes) increased approximately 3-fold. This positive trend may be attributable to various factors, such as improvements in organized rescue services, emergency medical management, and advancements in safety equipment. Research into the pathophysiology of avalanche burial and the optimization of medical treatment for those experiencing an avalanche have resulted in periodically updated international guidelines and checklists.^7,8,9^ First responders and search-and-rescue teams have been informed about these guidelines and trained in rescue techniques and first medical management. This concerted effort has likely played a significant role in advancing on-site management practices for individuals experiencing an avalanche. Groundbreaking medical device innovations within prehospital and in-hospital settings, such as mechanical chest compression devices and extracorporeal membrane oxygenation, have likely contributed to improving survival outcomes in long-term burial.^10,11^ Some recently developed artificial air pocket devices may also have improved long-term survival by delaying the onset of hypoxia and hypercapnia.^12,13^

This study found that the total survival rate among individuals critically buried by avalanche significantly increased over the past 4 decades. This improvement is primarily attributable to a considerable reduction in overall rescue time, which decreased from 45 to 25 minutes. Additionally, the median rescue time for individuals rescued by companions decreased from 15 to 10 minutes, closely aligning with the 10-minute onset of asphyxia. The reduced rescue time may be attributable to better education and training of winter sports enthusiasts, who now commonly adopt essential safety practices, such as carrying transceivers, probes, and shovels.^14^ Avalanche transceivers, electronic devices for the prompt location of individuals critically buried by avalanche, have been substantially improved in recent decades and have been associated with significantly reduced rescue times and mortality rates.^15^ Also, the accelerated dispatch of organized rescue teams to avalanche accident sites, facilitated by extended cell phone coverage in mountainous and remote areas and the standardized use of rescue helicopters, may have significantly enhanced professional rescue response times, shortened the duration of burial, and improved medical decision-making compared with 40 years ago.^16^ Nevertheless, in this study, when individuals were rescued by companions, their chances of survival were approximately 3 times higher compared with being rescued by organized rescue teams.

### Limitations

The main limitation of our study is the lack of data on the duration of burial for a substantial number of avalanche survivors and nonsurvivors. To address this, we used inverse transform sampling for imputation, applying it separately for each type of rescue and differentiating between survivors and nonsurvivors.

## Conclusions

This cohort study found that over the past 40 years, avalanche survival rates in Switzerland increased by 9.6 percentage points and the rescue time for individuals critically buried by avalanche decreased by 44.4%. Long-term survival also increased approximately 3-fold. These findings are likely attributable to a concerted effort by many stakeholders to improve avalanche search-and-rescue techniques and medical treatment. However, the findings suggest that the risk of early suffocation may begin earlier than previously assumed, and survival chances decreased to 31% for rescues between 10 and 30 minutes after burial. Asphyxia remains the leading cause of death from avalanches.

## Notes

Supplement 1.
eMethods.
**eTable.** Missing Rescue Times According to Rescue Type and Survival Status **eFigure 1.** Survival Functions With Imputed Missing Rescue Times **eFigure 2.** Survival Functions of Original and Imputed Data Using the Reference Survival Curve **eFigure 3.** z-Curve for the 1000 Single P Values Indicating That Most Tests Were Not Significant
eReference

Supplement 2.
Data Sharing Statement

## References

[1] Falk M, Brugger H, Adler-Kastner L. Avalanche survival chances. Nature. 1994;368(6466):21. doi:10.1038/368021a0 7969398

[2] Turnbull BW. Nonparametric estimation of a survivorship function with doubly censored data. J Am Stat Assoc. 1974;69(345):169-173. doi:10.1080/01621459.1974.10480146

[3] von Elm E, Altman DG, Egger M, Pocock SJ, Gøtzsche PC, Vandenbroucke JP; STROBE Initiative. The Strengthening the Reporting of Observational Studies in Epidemiology (STROBE) statement: guidelines for reporting observational studies. Lancet. 2007;370(9596):1453-1457. doi:10.1016/S0140-6736(07)61602-X 18064739

[4] Turnbull BW. The empirical distribution function with arbitrarily grouped, censored and truncated data. J R Stat Soc *Series B Stat Methodol*. 1976;38(3):290-295. doi:10.1111/j.2517-6161.1976.tb01597.x

[5] Haegeli P, Falk M, Brugger H, Etter HJ, Boyd J. Comparison of avalanche survival patterns in Canada and Switzerland. CMAJ. 2011;183(7):789-795. doi:10.1503/cmaj.101435 21422139 PMC3080528

[6] Strapazzon G, Paal P, Schweizer J, . Effects of snow properties on humans breathing into an artificial air pocket—an experimental field study. Sci Rep. 2017;7(1):17675. doi:10.1038/s41598-017-17960-4 29247235 PMC5732296

[7] Pasquier M, Strapazzon G, Kottmann A, . On-site treatment of avalanche victims: scoping review and 2023 recommendations of the International Commission for Mountain Emergency Medicine (ICAR MedCom). Resuscitation. 2023;184:109708. doi:10.1016/j.resuscitation.2023.109708 36709825

[8] Lott C, Truhlář A, Alfonzo A, ; ERC Special Circumstances Writing Group Collaborators. European Resuscitation Council guidelines 2021: cardiac arrest in special circumstances. Resuscitation. 2021;161:152-219. doi:10.1016/j.resuscitation.2021.02.011 33773826

[9] Kottmann A, Blancher M, Spichiger T, . The Avalanche Victim Resuscitation Checklist, a new concept for the management of avalanche victims. Resuscitation. 2015;91:e7-e8. doi:10.1016/j.resuscitation.2015.03.009 25796998

[10] Ruttmann E, Weissenbacher A, Ulmer H, . Prolonged extracorporeal membrane oxygenation-assisted support provides improved survival in hypothermic patients with cardiocirculatory arrest. J Thorac Cardiovasc Surg. 2007;134(3):594-600. doi:10.1016/j.jtcvs.2007.03.049 17723804

[11] Rauch S, Strapazzon G, Brodmann M, . Implementation of a mechanical CPR device in a physician staffed HEMS—a prospective observational study. Scand J Trauma Resusc Emerg Med. 2018;26(1):36. doi:10.1186/s13049-018-0503-4 29704898 PMC5923001

[12] Grissom CK, Radwin MI, Harmston CH, Hirshberg EL, Crowley TJ. Respiration during snow burial using an artificial air pocket. JAMA. 2000;283(17):2266-2271. doi:10.1001/jama.283.17.2266 10807386

[13] Strapazzon G, Rauch S, Malacrida S, . Comparative effectiveness of an artificial air pocket device to delay asphyxiation in supine individuals critically buried in avalanche debris. JAMA Netw Open. 2023;6(5):e2313376. doi:10.1001/jamanetworkopen.2023.13376 37184835 PMC12578492

[14] Procter E, Strapazzon G, Dal Cappello T, Castlunger L, Staffler HP, Brugger H. Adherence of backcountry winter recreationists to avalanche prevention and safety practices in northern Italy. Scand J Med Sci Sports. 2014;24(5):823-829. doi:10.1111/sms.12094 23815413

[15] Brugger H, Etter HJ, Zweifel B, . The impact of avalanche rescue devices on survival. Resuscitation. 2007;75(3):476-483. doi:10.1016/j.resuscitation.2007.06.002 17689170

[16] Strapazzon G, Plankensteiner J, Mair P, . Prehospital management and outcome of avalanche patients with out-of-hospital cardiac arrest: a retrospective study in Tyrol, Austria. Eur J Emerg Med. 2017;24(6):398-403. doi:10.1097/MEJ.0000000000000390 26990382

