# Supplementary material for: Avalanche Survival Rates in Switzerland, 1981-2020
Source: JAMA Netw Open. 2024 Sep 25;7(9):e2435253. doi: 10.1001/jamanetworkopen.2024.35253 (PMC11425148; doi:10.1001/jamanetworkopen.2024.35253)
Supplement: Supplement 1. — eMethods. eTable. Missing Rescue Times According to Rescue Type and Survival Status eFigure 1. Survival Functions With Imputed Missing Rescue Times eFigure 2. Survival Functions of Original and Imputed Data Using the Reference Survival Curve eFigure 3. z-Curve for the 1000 Single P Values Indicating That Most Tests Were Not Significant eReference [file jamanetwopen-e2435253-s001.pdf]

Supplementary Online Content

Rauch S, Brugger H, Falk M, et al. Avalanche survival rates in Switzerland, 1981-2020. *JAMA Netw Open*. 2024;7(9):e2435253. doi:10.1001/jamanetworkopen.2024.35253

eMethods

eTable. Missing Rescue Times According to Rescue Type and Survival Status

eFigure 1. Survival Functions With Imputed Missing Rescue Times

eFigure 2. Survival Functions of Original and Imputed Data Using the Reference Survival Curve

eFigure 3. z-Curve for the 1000 Single *P* Values Indicating That Most Tests Were Not Significant

eReference

This supplementary material has been provided by the authors to give readers additional information about their work.

© 2024 Rauch S et al. *JAMA Network Open*.

eMethods

Survival analyses were performed using R, version 4.4.1 (R Group for Statistical Computing), and its packages survival, interval, survminer, and icenReg. Victims extricated alive are right-censored at the time of extrication, whereas non-survivors are left-censored, with death occurring somewhere between zero and the time of extrication.

The number of missing burial times has increased in recent years to about 22%, mostly among survivors (90%, see Table 1 of the article). To obtain an unbiased estimate of the survival function, it is necessary to impute the missing rescue times. The distribution of rescue time depends on the type of rescue (organized vs. companions or self-rescue) and survival status (survivors vs. non-survivors). Therefore, missing rescue times were imputed via inverse transform sampling [1], conducted separately for each rescue type and distinct for survivors/non-survivors. For every combination of rescue type and survival status, 10 in total (see eTable 1), a respective number of random values were drawn from a uniform distribution in the range of 0 to 1. For every value, the percentile of the respective cumulative distribution of rescue times was then used as the imputed rescue time, 253 imputed values in total per run.

For each of the 1,000 runs (imputed datasets), we calculated the survival function and used the median value at each time point over the 1,000 individual values as the reference survival. Furthermore, at each time point, we calculated the squared difference from this reference survival to each individual value and summed them up over all time points for each individual survival function. The survival function with the lowest sum was then used as the reference survival function, and the respective 253 imputed rescue times were subsequently used for further analysis (eFig2).

With original data, survival distributions per decade (1981-1990, 1991-2000, 2001-2010, 2011-2020) were significantly different (interval::icens p = .011), whereas with imputed data, they were not (icens, p = .10). Furthermore, parameter estimates from the proportional hazard model of function icenReg::ic_sp for the periods 1991-2000, 2001-2010, 2011-2020 were -0.212 (p = .120), -0.003 (p = .985), 0.280 (p = .024) with original data and -0.230 (p = .087), -0.163 (p = .187), 0.058 (p = .619) with imputed data, indicating that the significant effect for the decade 2011-2020 is not present when data is imputed (p = .024 vs. p = .619).

© 2024 Rauch S et al. *JAMA Network Open*.

As a sensitivity analysis, we further calculated the parameter estimate for the decade 2011-2020 with the respective p-value for each imputed dataset, giving us 1,000 individual p-values. The respective z-curve shows that there is no evidence of a significant effect, as the distribution of the z-scores spans values below the threshold of 1.96 (eFig3).

© 2024 Rauch S et al. *JAMA Network Open*.

eTable: Missing rescue times according to rescue type and survival status

Rescue type	survivors	non-survivors	Total
unknown	40	3	43
Rescue by companions	147	6	153
Organized rescue service	19	15	34
Self-rescue	23	0	23
Total	229	24	253

© 2024 Rauch S et al. *JAMA Network Open*.

eFigure 1: Survival functions (n=1000) with imputed missing rescue times. Black represents the estimated survival function using only valid data, red represents the median survival at each time point from the survival functions with imputed data (reference survival), while green represents the reference survival function with lowest distance to the reference survival.

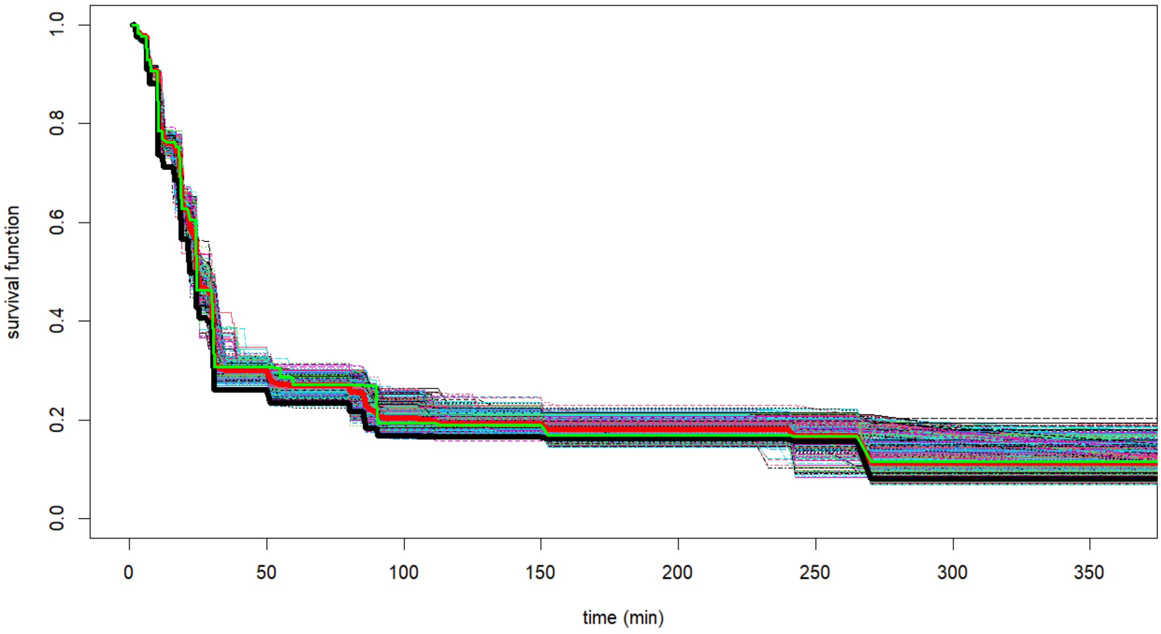

© 2024 Rauch S et al. *JAMA Network Open*.

eFigure 2: Survival functions of original and imputed data using the reference survival curve

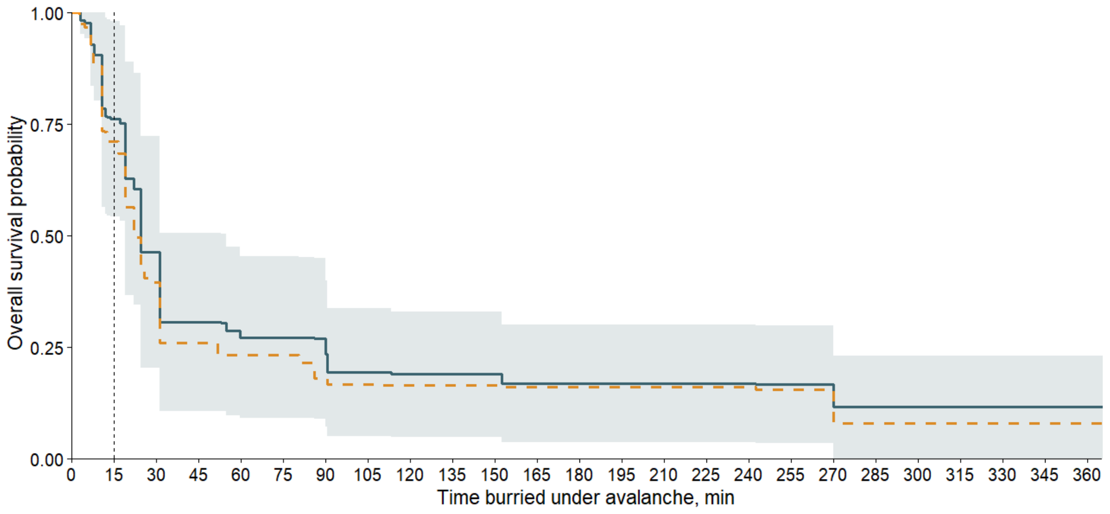

No. at risk

	0	15	30	45	60	75	90	105	120	135	150	165	180	195	210	225	240	255	270	285	300	315	330	345	360	375
CH 1981-2020 imputed	1643	849	380	195	138	123	94	68	62	58	56	47	46	43	42	39	38	37	36	25	25	25	25	25	25	24
CH 1981-2020	1390	710	301	152	113	102	70	57	52	49	47	43	43	40	39	36	35	33	32	17	17	17	17	17	17	17

Time burried under avalanche, min

© 2024 Rauch S et al. *JAMA Network Open*.

eFigure 3: z-curve for the 1000 single p-values indicating that most tests were not significant

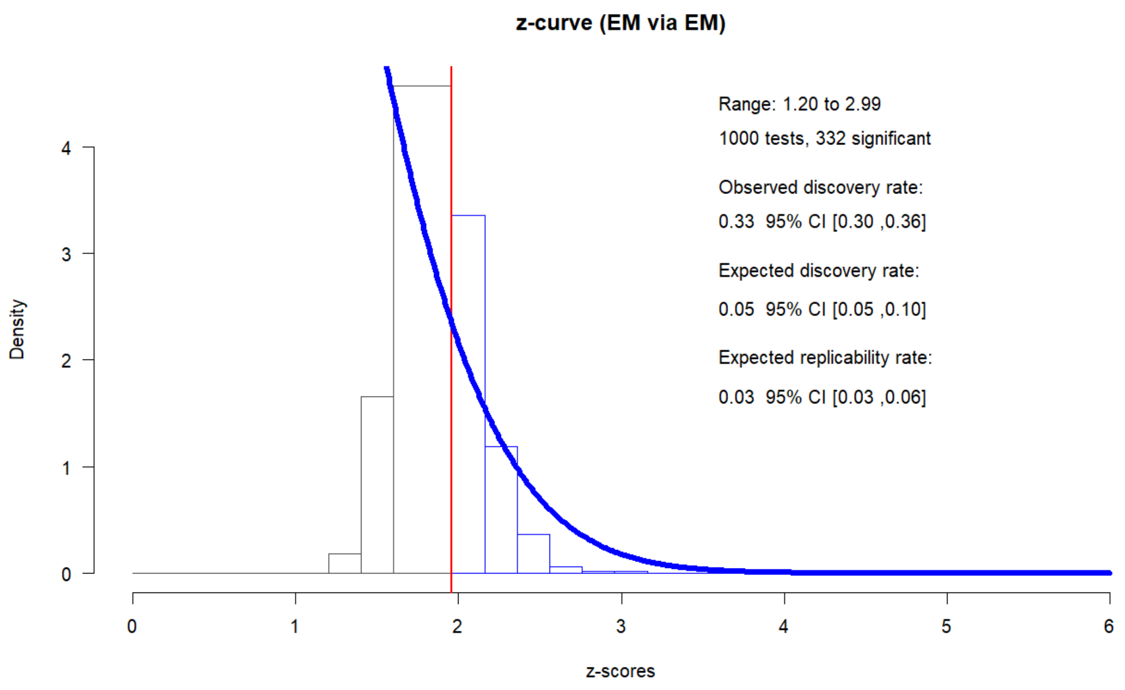

© 2024 Rauch S et al. *JAMA Network Open*.

eReference

[1] Devroye L, Non-Uniform Random Variate Generation. Springer New York, NY, 2013. https://doi.org/10.1007/978-1-4613-8643-8

© 2024 Rauch S et al. *JAMA Network Open*.
