# Supplementary material for: Avalanche Survival Rates in Switzerland, 1981-2020
Source: JAMA Netw Open. 2024 Sep 25;7(9):e2435253. doi: 10.1001/jamanetworkopen.2024.35253 (PMC11425148; doi:10.1001/jamanetworkopen.2024.35253)
Supplement: Supplement 2. — Data Sharing Statement [file jamanetwopen-e2435253-s002.pdf]

Data Sharing Statement

Rauch. Avalanche Survival Rates in Switzerland, 1981-2020. *JAMA Netw Open*. Published September 25, 2024. doi:10.1001/jamanetworkopen.2024.35253

Data
Data available: No
